# Comprehensive diagnosis and treatment of alveolar echinococcosis: A single-center, long-term observational study of 312 patients in Germany

**DOI:** 10.3205/id000027

**Published:** 2017-01-06

**Authors:** Beate Grüner, Petra Kern, Benjamin Mayer, Tilmann Gräter, Andreas Hillenbrand, Thomas E. F. Barth, Rainer Muche, Doris Henne-Bruns, Wolfgang Kratzer, Peter Kern

**Affiliations:** 1 Department of Internal Medicine III, University Hospital Ulm, Germany; 2 Institute of Epidemiology and Medical Biometry, University of Ulm, Germany; 3 Department of Interventional and Diagnostic Radiology, University Hospital Ulm, Germany; 4 Department of Surgery I, University Hospital Ulm, Germany; 5 Institute of Pathology, University Hospital Ulm, Germany; 6 Department of Internal Medicine I, University Hospital Ulm, Germany

**Keywords:** alveolar echinococcosis, Echinococcus multilocularis, antiparasitic treatment, surgery, classification, risk factors

## Abstract

Alveolar echinococcosis (AE) is the most Iethal human helminthic infection. The malignancy-like disease is rare, but morbidity and treatment costs are high. Objective of the study was to identify factors at baseline and during specific AE therapy influencing the long-term outcome of the disease.

All patients with AE seen at the specialized treatment unit in Ulm between January 1992 and December 2011 were included in the analysis.

The data of 312 patients were analyzed; 108 were diagnosed before 2000 (series A), 204 since 2000 (series B); 290 received specific AE treatment. Patients of series B were more often symptom-free at diagnosis (44.1% vs. 21.3%), had lower disease stages (50.0% vs. 34.2%) and more complete resections (57.7% vs. 20.0%), but higher rates of side effects and drug toxicity (54.1% vs. 40.8%). In series B, more patients remained relapse- or progression-free after 5 years (90.5% vs. 82.8%); after 10 years, the ratio of relapses converged (70.3% vs. 66.9%, p=0.0507). Relapses or progression occurred more often after incomplete surgery or long treatment pauses. The 5-year and 10-year survival rates were 96.9% and 90.6%, respectively, and 17% of the patients were cured.

We observed a shift towards early diagnosis, earlier initiation of specific therapy and more complete resections after 2000. Although diagnosis and treatment of AE pose a challenge, with an individual interdisciplinary management 88.8% of the patients have a favorable outcome.

## Introduction

Human alveolar echinococcosis (AE) is an orphan zoonosis caused by the larval stage of the fox tapeworm *Echinococcus multilocularis*. AE primarily affects the liver, as a cancer-like lesion, often infiltrates neighboring organs and can eventually metastasize [1], [2]. AE prognosis was poor until the 1970s [3]; since the 1980s, benzimidazole therapy (BMZ) improved survival rates [4]. Today, large case series from Switzerland and France report good prognoses [5], [6]. 

Radical surgery followed by a 2-year BMZ administration is the standard treatment aiming at cure, but the majority of patients is inoperable and needs long-term BMZ treatment [2], [7]. The consensus recommendation of the WHO Informal Working Group on Echinococcosis (IWGE [8]) suggests that patients should be treated in specialized centers by multidisciplinary teams.

Human AE is emerging in Europe, increasing in known and spreading to new foci [9], [10], [11], [12], [13], [14]. If the trend persists, more human cases can be expected in the near future and pose a challenge to specialized health care. The aim of the study was to assess survival, progression and relapse rates of AE depending on the period of diagnosis, the severity of the disease at diagnosis, and on stage-adapted therapy.

## Patients and methods

### Diagnosis and definition of AE

The Ulm Working Group on Echinococcosis was established in 1991 at Ulm University Hospital, a tertiary care center. The study includes all AE patients seen at this unit between January 1992 and December 2011. Follow-up ended on 12/31/2012.

At their first visit, patients had either findings suggestive of AE or were already under treatment for manifest AE. The diagnosis was ascertained by:

- abdominal ultrasound (US), computed tomography (CT) or magnetic resonance imaging (MRI);
- immunodiagnosis with standard commercial tests, i.e. indirect hemagglutination (IHA: Cellognost Echinococcosis, Dade Behring, Germany), enzyme-linked immunosorbent assay (*Echinococcus multilocularis* – ELISA Em2plus, Nr. 9300, Bordier Affinity, Crissier, Switzerland);
- confirmatory serology with crude antigens, Em10, and antigen B (performed at the national reference laboratory for echinococcosis, Institute of Microbiology and Hygiene, Wuerzburg, Germany);
- histopathology with haematoxylin-eosin staining, PAS and immunohistological staining (EM2G11; [15]).

Since 2000, ^18^F-fluoro-desoxyglucose positron emission tomography (FDG-PET) was applied. FDG enrichment around AE lesions is interpreted as a larval metabolic activity [16]. 

### Classification

According to the WHO-IWGE criteria, since 2000 the certainty of the first diagnosis was classified as “possible” (clinical presentation AND epidemiological history AND imaging findings OR serology positive for AE), “probable” (clinical presentation AND epidemiological history AND imaging findings AND serology positive for AE) or “confirmed” (the above AND histopathology compatible with AE AND/OR *E. multilocularis*-nucleic acid sequences in a clinical specimen) [8], [17]. Location and size of the larval lesions at diagnosis were documented using the PNM classification [18], a modified TNM classification (P0-4, parasitic mass in the liver; N0-1, involvement of neighboring organs; M0-1, metastases).

### Treatment and follow-up

Treatment followed the WHO-IWGE recommendations [8]. After liver resection, surgical specimen were graded as complete (R0) or incomplete resection (R1, R2). The patients received BMZ, either albendazole (ABZ) or mebendazole (MBZ). Blood chemistry, hematology and immunodiagnostic tests were performed regularly and ABZ sulfoxide plasma levels were obtained [19], [20]. Treatment efficacy was monitored by US examination and, every 24 months, by alternating FDG-PET/CT scans and MRI. Side effects of BMZ therapy were recorded throughout follow-up. In case of drug toxicity, medication was stopped and re-exposure started with a lower dosage. If the toxicity remained, the patients were switched to the other BMZ, or received experimental drugs [21], [22], [23] or had prolonged medication-free periods. Drug-free intervals due to other reasons were self-reported by the patients. 

Structured treatment interruption (STI; [24]) was a goal for patients with an N0M0 status at diagnosis, after surgery and a 2-year BMZ therapy with negative FDG-PET scans. For patients with non-resectable lesions strict preconditions had to be fulfilled for an STI (Table 1 (Tab. 1)). If FDG-PET reverted to positive results, BMZ therapy was restarted.

### Data assessment, definitions and analysis

Data were assessed retrospectively from the unit’s medical charts, the records of other hospitals and family doctors. We recorded age, sex, date of diagnosis, symptoms, comorbidities and PNM stage at first diagnosis; start of specific treatment, type of treatment, all drugs used, drug switches, side effects, periods of abrogated medication, relapses or progression of larval lesions and their dates, date of death.

For the purposes of this study we made the following a priori assumptions: the start of any specific AE therapy was “early” when it occurred during the first 2 years after diagnosis. Non-adherence to continuous BMZ therapy, whether due to drug intolerance, misunderstandings or non-compliance, was noted as “pause”, if it lasted longer than 3 months. Shorter drug-free periods were regarded as not clinically significant. After an STI, cure was assumed for patients with surgery when parasitic lesions remained undetectable during the 2 years following BMZ interruption. Cases with BMZ treatment alone were not defined as cured. The patient cohort was stratified into series A containing diagnoses until December 1999 and series B from January 2000 onwards. This date was chosen as patients diagnosed since 2000 benefit, immediately after diagnosis, from improved imaging techniques and serological markers, and the application of the PNM staging system which guides treatment decisions according to the updated WHO-IWGE recommendations.

Statistics were done with SAS software version 9.3. Outcome parameters were: overall survival after diagnosis; relapse after surgery or progression of remaining liver lesions in size or new lesions in other organs; time to relapse or progression after start of AE-specific treatment. Survival was estimated by the Kaplan-Meier method. Covariates which may impact the time until relapse or progression entered univariate Cox proportional hazard regression models initially, for which hazard ratios (HR) with corresponding 95% confidence intervals (CI) were calculated. Variables with a p-value ≤0.1 entered the multivariable model; model fit was assessed with Akaike’s Information Criterion (AIC).

## Results

A total of 312 AE patients was included; 35% were diagnosed until 1999 (case series A), 65% since 2000 (series B). Their median age at first diagnosis was 51.2 years (Table 2 (Tab. 2)). Only 10% of all cases were younger than 25 years; 8 were children or adolescents. Gender distribution showed a preponderance of women; the female:male ratio was slightly higher in series A than B. In series B, 54.9% of the diagnoses were rated “confirmed”, 35.8% “probable”, and 9.3% “possible”.

One third of all patients had parasitic lesions in neighboring organs (N1) of the liver, 10% had distant metastases (M1). 4 cases had extrahepatic AE only (spleen, 2; vertebra, 1; retroperitoneal/kidney, 1; P0, Table 3 (Tab. 3)). “Spleen” cases were classified as stage I (localized disease), other extrahepatic cases as stage IV (extended disease). Cases from series B more often presented with stage I lesions at diagnosis than patients from series A (12.8% vs. 6.5%) and had more frequently lesions defined as localized disease (stage I to IIIa; 50.0% vs. 34.3%, Table 3 (Tab. 3)).

“Inactive” lesions compatible with an aborted infection were identified in 17 individuals; they remained untreated, and were invited for regular medical checks (Table 4 (Tab. 4)). Notably, the majority had presented with status “possible” AE; two of them reverted later to “active” disease and received BMZ.

Specific and unspecific symptoms were less frequent in case series B (44.6%) than in series A (61.1%; Table 2 (Tab. 2)). The occurrence of any kind of symptoms was closely associated with the disease stage: rates of patients with symptoms increased from 24.2% (stage I) to 32.6% (stage II), 50.8% (stage IIIa), 54.4% (stage IIIb) and 68.0% at stage IV. More cases in series A had no concomitant health problems when first diagnosed with AE (76.9% vs. 45.6%, Table 2 (Tab. 2)). During follow-up, 10.2% of the patients in series A were affected by malignancies; 3.4% in series B suffered from newly diagnosed chronic inflammatory diseases.

The ratio of patients undergoing liver surgery followed by postoperative BMZ compared to those receiving BMZ alone, was higher in case series A (50.9% vs. 38.2%, Table 5 (Tab. 5)). The median time from diagnosis to surgery did not differ between the two patient series. In series B a higher proportion of cases was operated at stage I, and conversely, a higher proportion of cases in series A at stage IV. R0 resections were achieved for 53.8% in series B compared to 20% in series A. Incomplete surgery was more often reported in series A (70.9%) than in series B (42.3%).

Biliary complications requiring interventions such as stents or drainages occurred more often in series A than series B (Table 5 (Tab. 5)) and were more frequent in patients with surgery than cases with BMZ therapy alone (21.1% vs. 13.4%).

Almost all patients of series B, and 76.7% of the cases of series A, received specific BMZ treatment early after diagnosis (≤2 years; Table 6 (Tab. 6)). In series A, 15 patients had had to wait for >10 years since BMZ were not routinely applied before 1981.

BMZ effects were assessed for 288 patients (Table 6 (Tab. 6)). The majority of them (58.7%) took only one of the BMZ, others had to be switched once or twice. 45.5% did not experience any side effects of long-term BMZ treatment; 28.5% suffered from elevated liver transaminases, 15% reported abdominal discomfort or diarrhea, 6.9% had severe liver toxicity requiring drug switches or pauses, 7.0% had leukopenia and 9% skin-related problems. Most patients (224; 77.8%) had an uninterrupted BMZ treatment. Pauses due to either side-effects or non-compliance were reported for 21.4% of patients from series A and 9.6% from series B.

During follow-up, 27 cases had had an intermittent STI of BMZ therapy, but after 18 months, FDG enrichment was seen in PET-CT. BMZ was thus restarted for these patients. At the end of follow-up, medical treatment was stopped in 85 of 288 patients (29.5%); 68 had had surgery, 17 had been treated with BMZ alone (Table 7 (Tab. 7)). Of the patients with surgery, 49 (17.0% of the whole cohort) were regarded as potentially cured of AE; for 19 the observation period after STI was still too short to judge or they had a R2 surgery; they were rated as “stable” (Table 8 (Tab. 8)). In 8 of the cured patients, BMZ treatment had been <1.75 years due to either BMZ toxicity or non-compliance; their follow-up after STI ranged from 3.8 to 10.4 years. Although they did not complete the recommended duration for postsurgical BMZ prophylaxis, they were regarded as cured, since their follow-up after surgery ranged from 4.5 to 11.1 years and lesions remained undiscernible during this period. Overall, 228 patients (79.2%) had a stable course of the disease (Table 8 (Tab. 8)). In 16 cases, the lesions were progressive (5.6%), 19 could not be rated after their first visit to the center (6.1%).

The study includes patients with long case histories, the longest survival after diagnosis was 47.6 years. 30 persons (9.6%) had histories of >25 years. 40 patients died during follow-up; 15 deaths (37.5%) were associated with the disease (Table 8 (Tab. 8)), causes of death were unrelated to AE for 19 patients (47.5%) or unknown (6 cases). The 5-year and 10-year survival rates were 96.9% and 90.6%, respectively.

**Figure 1:**
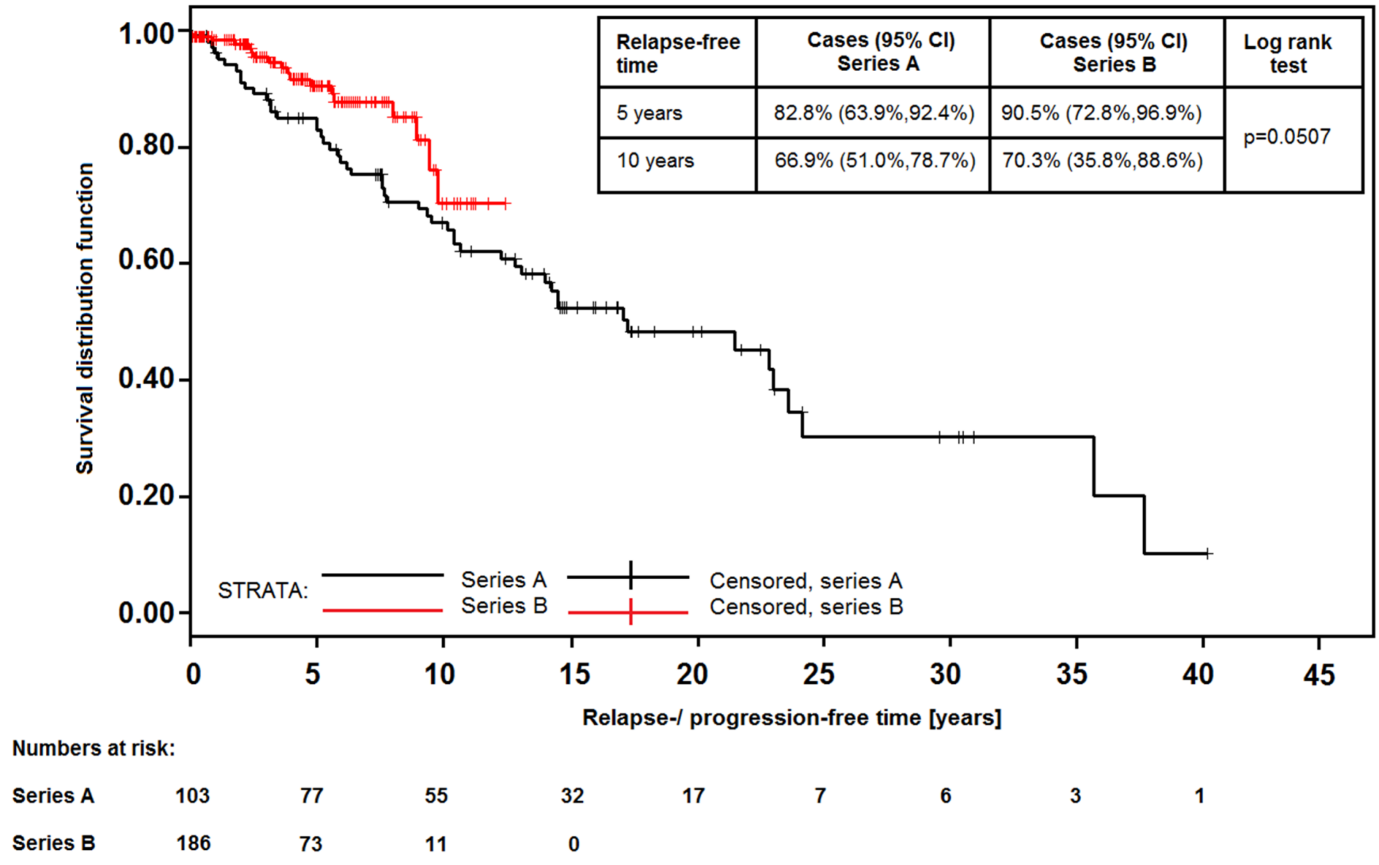
Relapse-free time after the start of specific treatment for AE (either BMZ treatment or first liver surgery; CI=Hall-Wellner confidence limits)

69 of all patients under treatment experienced progression of residual lesions or relapses after surgery (Table 8 (Tab. 8)); the number was higher in series A (49.5%) than in series B (9.6%). 4 of the patients in series A had continuous progressive disease; 17 patients in series A and 1 patient in series B had 2 or 3 relapses. Progression was seen more frequently in cases who received specific treatments “late” (>2 years) after diagnosis (71.4%), in contrast to those with an immediate start of treatment (either surgery or BMZ alone, 17.6%). In 2 patients progression occurred during STI, one case relapsed 4 years after abrogation. Patients from series B had a better chance to stay relapse-free after 5 years (90.5% vs. 82.8%), but the advantage was less after 10 years (70.3% vs. 66.9%; Figure 1 (Fig. 1)).

Univariate cox regression models showed some beneficial trend of the probability of relapses in patients with low PNM stages (p=0.080) and a significant reduction in cases without distant metastases (p=0.029, Table 9 (Tab. 9)). The early onset of therapy after diagnosis more than halved the risk of relapse (HR=0.38, 95% CI 0.20–0.75, p=0.005), and likewise uninterrupted medication was associated with a significant reduction of the risk of a relapse (HR=0.43, 95% CI 0.26–0.71, p=0.001). The duration of medical pauses raised the probability of a relapse by 40.9%, adding up pauses and STI did not further increase the risk (38.9%). In multivariable analysis, which showed the best model fit (AIC 489.8), the early start of a specific therapy (p=0.011) as well as diagnosis after 2000 were found significant protective covariates, whereas prolonged duration of pauses or STI (p=0.025) increased the risk for the occurrence of relapses significantly (Table 9 (Tab. 9)).

## Discussion

A key feature of this study is the comprehensive follow-up of AE cases in a single center, including all diagnostic procedures. At the end of the follow-up period, 88.8% of the patients had a favorable outcome, were either cured by surgery or remaining lesions were rated as stable or regressive with BMZ treatment. This positive trend corroborates observations in Swiss and French patient cohorts [5], [6], [10], [25]. In addition, we identified the factors for longer relapse/progression-free survival: early start of (any) specific treatment, low PNM stage (I–IIIa) of AE at time of diagnosis, especially absence of metastases, and absence of chronic inflammatory diseases.

Patients diagnosed since 2000 had noticeable advantages compared to previous cases: less advanced disease (more N0M0 stages), less symptoms at diagnosis, earlier start of treatment, more complete resections and significantly less debulking surgery at advanced stages of AE. These factors were shown to impact the frequency and time of relapses after liver surgery or regrowth of inoperable lesions. The differences may be attributed to adherence to the treatment recommendations released by the WHO-IWGE [2], [8]. Since 2000, outcomes may have been improved also by a raised awareness of the disease among general practitioners, leading to less diagnoses by chance, and improved infrastructure with a low staff turnover rate in the treatment unit, leading to closer ties to patients and their doctors.

Recent case studies used the PNM staging system to delineate the severity of AE disease [6], [25], [26], [27]. The liver is the primary site of infection of *E. multilocularis* larva, but more than one third of the cases had lesions in other organs or tissues as well. Primary extrahepatic lesions were rare; we cared for four patients with spleen, vertebral or retroperitoneal lesions (1.2% as compared to the French series with 4.0% [6]). In case series B, the percentages of cases in the five PNM categories increased steadily from lower stage I to more severe stage IV, which shows that an assumed earlier diagnosis in recent years did not substantially reduce the number of advanced disease stages. This trend was also noted in other European cohorts [6], [28]. 

Diagnosis of AE still remains a challenge – even for experienced teams – as “confirmed” diagnosis was obtained for 55% of patients only, a “probable” diagnosis for 36%. For the latter, serological evidence was conclusive to diagnose AE with some certainty [8], [17]. However, nearly 10% of the cases fall into the category of “possible” diagnosis. A similar finding was reported in the French cohort underlining the difficulties of clear cut diagnostic findings compatible with AE. In our study, 2 of 17 patients with a “possible” diagnosis and classified as „inactive“ lesions compatible with aborted infection [29] reverted after years into an active disease necessitating BMZ treatment. We conclude that patients with an inactive disease require regular monitoring for many years including the use of FDG-PET/CTs for a precise activity evaluation of the larval tissue and a new ultrasonographic classification scheme [25], [26], [30], [31].

Treatment with BMZ was initiated 40 years ago [3]. Due to its pharmacokinetic properties albendazole became the drug of choice. Cyclic treatment was recommended [32], but we switched to a continuous medication for AE patients who could not be radically operated. Almost 50% experienced side-effects, mostly minor and transient, which could be managed with short medication pauses [17]. But 6.9% suffered from severe hepatotoxic effects necessitating long BMZ treatment pauses. Within our interdisciplinary network these cases were managed according to their individual needs, and some received experimental drugs (e.g. interferon-gamma, amphothericin, nitazoxanide) [21], [22], [23]. AE patients with chronic concomitant diseases accounted for 13.2%; the French study group reported 9.8% [33]. Their management may be difficult since treatment for their second main disorder can imply interactions with ABZ therapy.

Treatment options changed gradually during the long follow-up for patients in series A, whereas patients in series B benefit from improved therapeutic modalities right after diagnosis [2]. Our results point to the importance of an early start of appropriate treatment. The lack of any advantage of incomplete liver surgery on the long-term outcome was also proven by the Swiss cohort study [27], [28].

In series B, a lower rate of relapses 5 years after the start of a specific treatment may also be a result of improved patient management. It could, however, be an artifact due to shorter follow-up in this group. Since relapse rates rise again after 10 years, we assume that these patients were surveyed more strictly and that new or regrowing lesions were detected earlier. The retrospective nature of the database, high numbers of missing data in series A, and varied data quality from different sources (clinics and practices) are the main limitations of this study. Self-reported drug adherence may contain too optimistic values and underestimate the risk connected to medical pauses. The risk for biased parameter estimates due to retrospective data assessment was adequately addressed by applying a multivariable model.

Our study shows that for AE patients adherence to BMZ treatment for several years is crucial for obtaining long relapse-free periods or even cure. If unavoidable, drug-free intervals should be kept as short as possible. Doctors must respond to manifold reasons for such pauses; especially patients with long AE histories may be prone to non-compliance and overestimate side-effects.

## Abbreviations

- ABZ: albendazole
- AE: alveolar echinococcosis
- AIC: Akaike’s Information Criterion
- BMZ: benzimidazoles
- CI: confidence interval
- CT: computed tomography
- ELISA: enzyme-linked immunosorbent assay
- FDG-PET: ^18^F-fluoro-desoxyglucose positron emission tomography
- HR: hazard ratio
- IHA: indirect hemagglutination
- MBZ: mebendazole
- MRI: magnetic resonance imaging
- PAS: periodic acid-Schiff stain
- PNM: staging system for human AE with P0-4 = parasitic mass in the liver; N0-1 = involvement of neighboring organs; M0-1 = metastases
- R0: no residues at resection margins after surgical resection
- R1: microscopic residues after surgical resection
- R2: macroscopic residues after surgical resection
- STI: structured treatment interruption
- TNM: staging system for malignant tumors
- US: ultrasound
- WHO-IWGE: World Health Organization – Informal Working Group on Echinococcosis

## Notes

### Acknowledgements

We sincerely thank all patients and their physicians for providing information and for their persistent confidence in our work. We also would like to thank the many hospital doctors who cared for the patients during the long observation period. The assistance of former and current members of the Ulm Working Group on Echinococcosis is highly appreciated: Ambros Beer, Meinrad Beer, Klaus Brehm, Andreas K. Buck, Klaus Buttenschoen, Daniela Carli-Buttenschoen, Martina Furitsch, Mark M. Haenle, Georg Haerter, Nils P. Krochmann, Sabine Kurz, Sven N. Reske, Stefan Reuter, Thomas Romig, Peter Soboslay, Dennis Tappe, and Suemeyra Tasdemir.

### Competing interests

The authors declare that they have no competing interests.

### Financial support

The final statistical work-up of the study was supported by the DFG (Ke 282/8). The early epidemiological part (European Echinococcosis Registry) was funded by a pilot program of the European Commission (Directorate General V), and in part by the Paul-Ehrlich-Society of Chemotherapy (PEG). The sponsors had no role in study design, collection, analysis or interpretation of data, nor in the writing or in the decision to submit the article for publication.

### Ethics

The study was approved by the ethical committee of Ulm University (166/13) and performed according to the declaration of Helsinki; patients gave informed consent to use their data for study purposes.

### Trial registration

ClinicalTrials.gov ID: NCT02509884 

## Figures and Tables

**Table 1:**
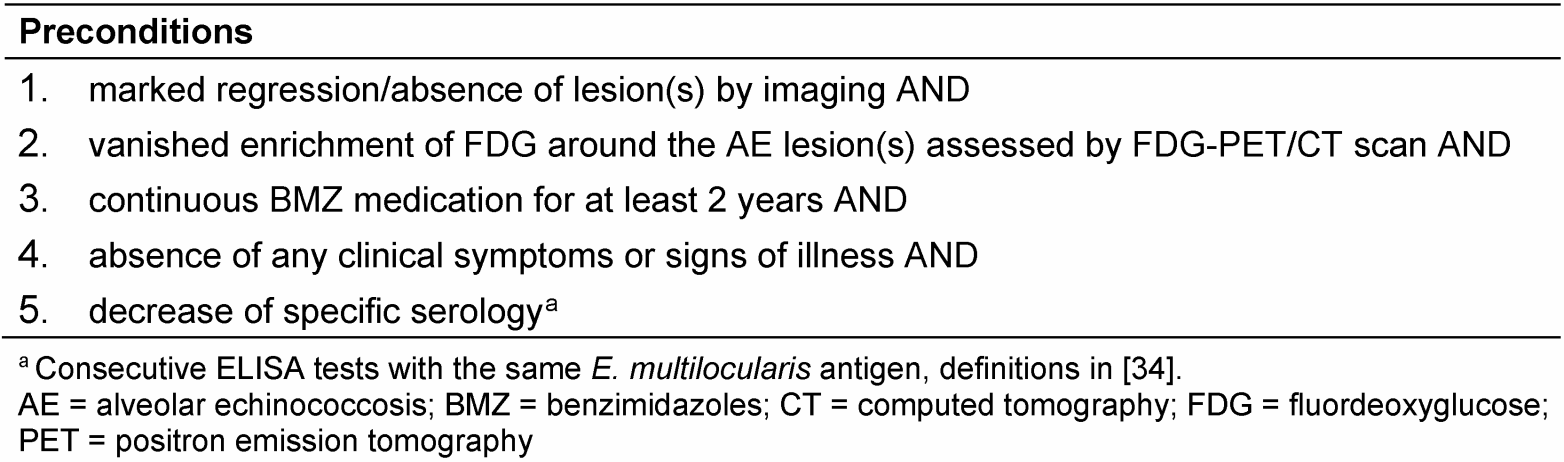
Preconditions for structured treatment interruption (STI) for patients with non-resectable lesions and no evidence of extrahepatic lesions

**Table 2:**
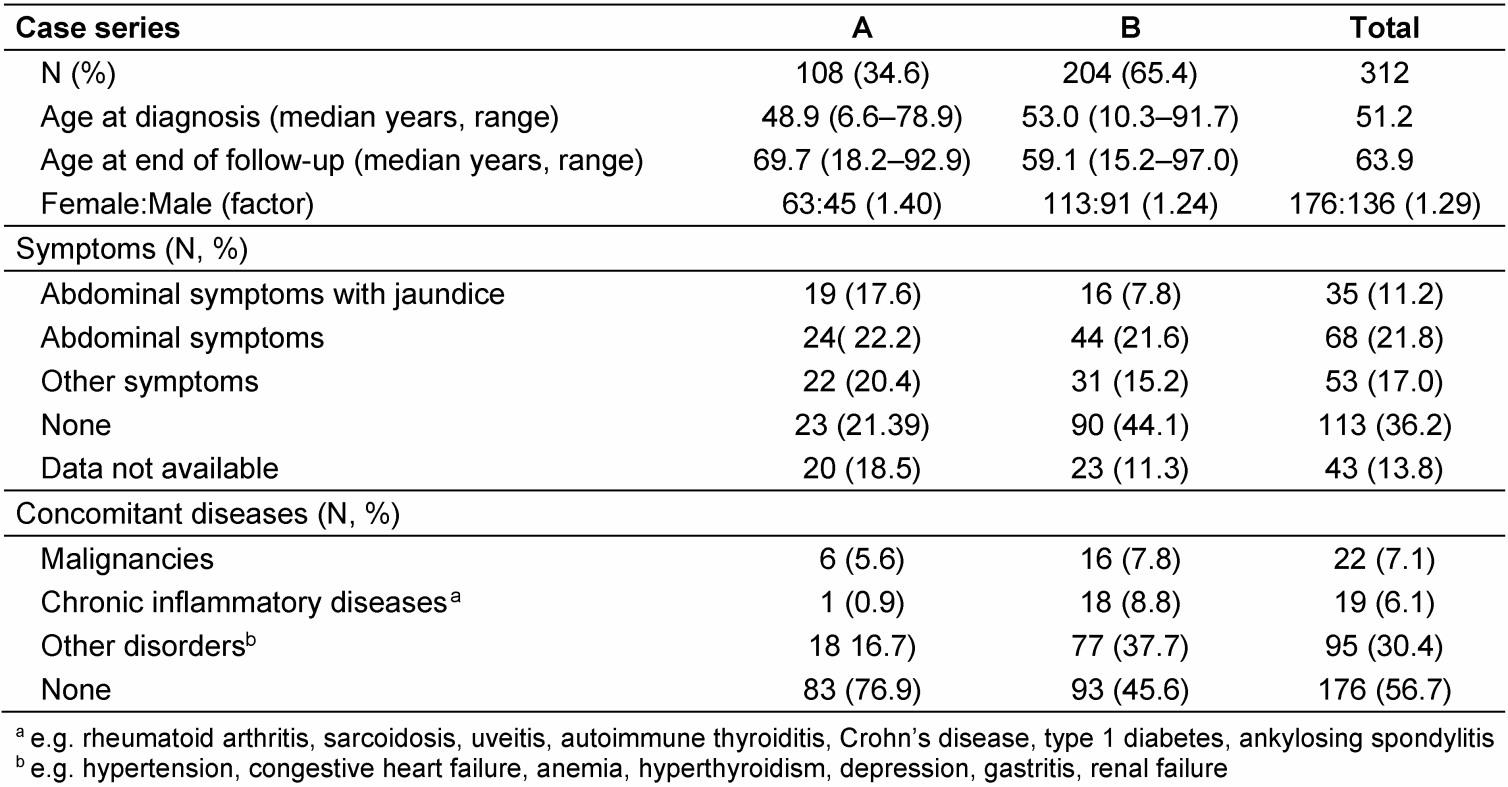
Demographic data, symptoms and concomitant diseases at first diagnosis

**Table 3:**
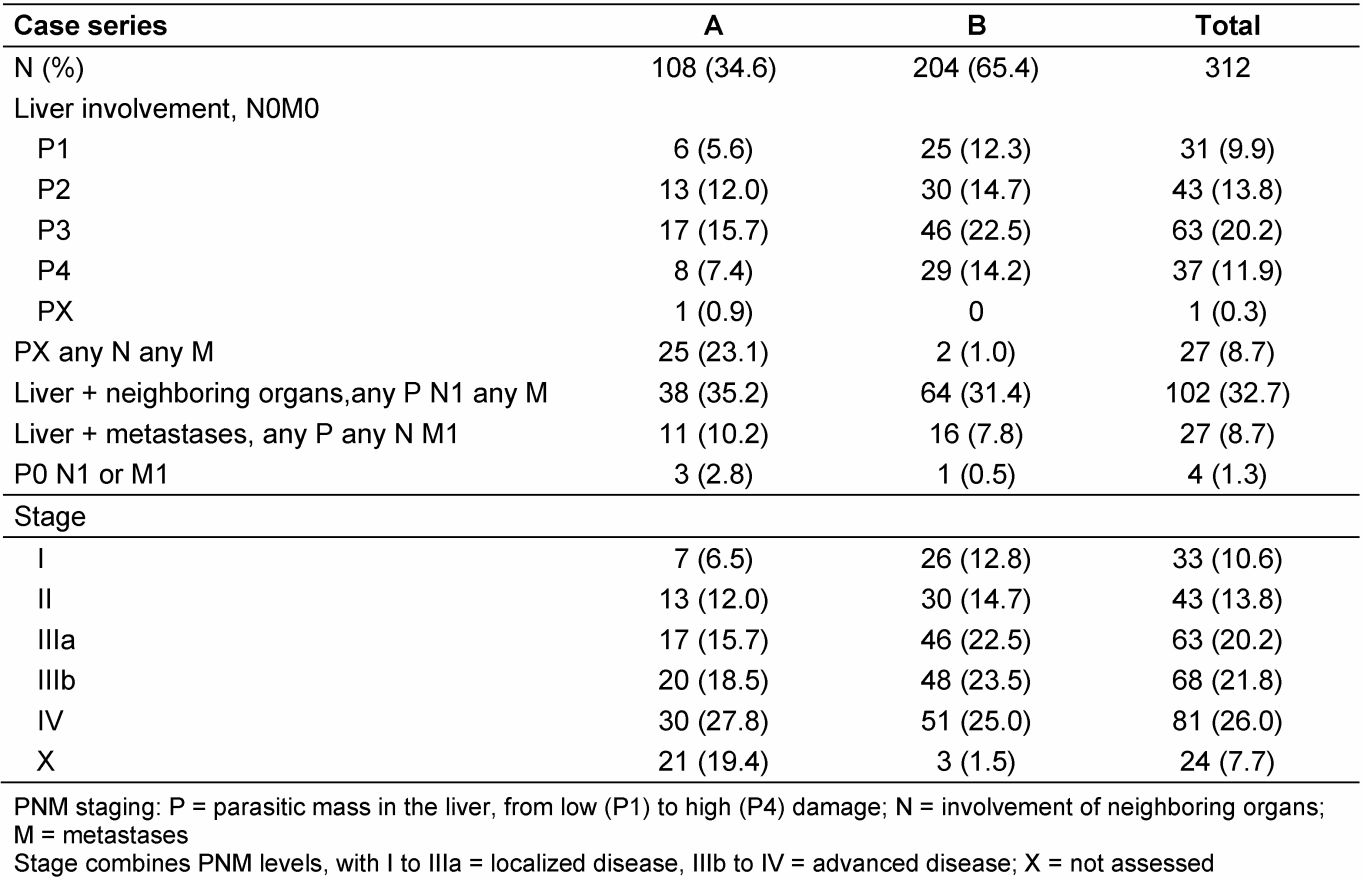
Classification and PNM staging of alveolar echinococcosis

**Table 4:**
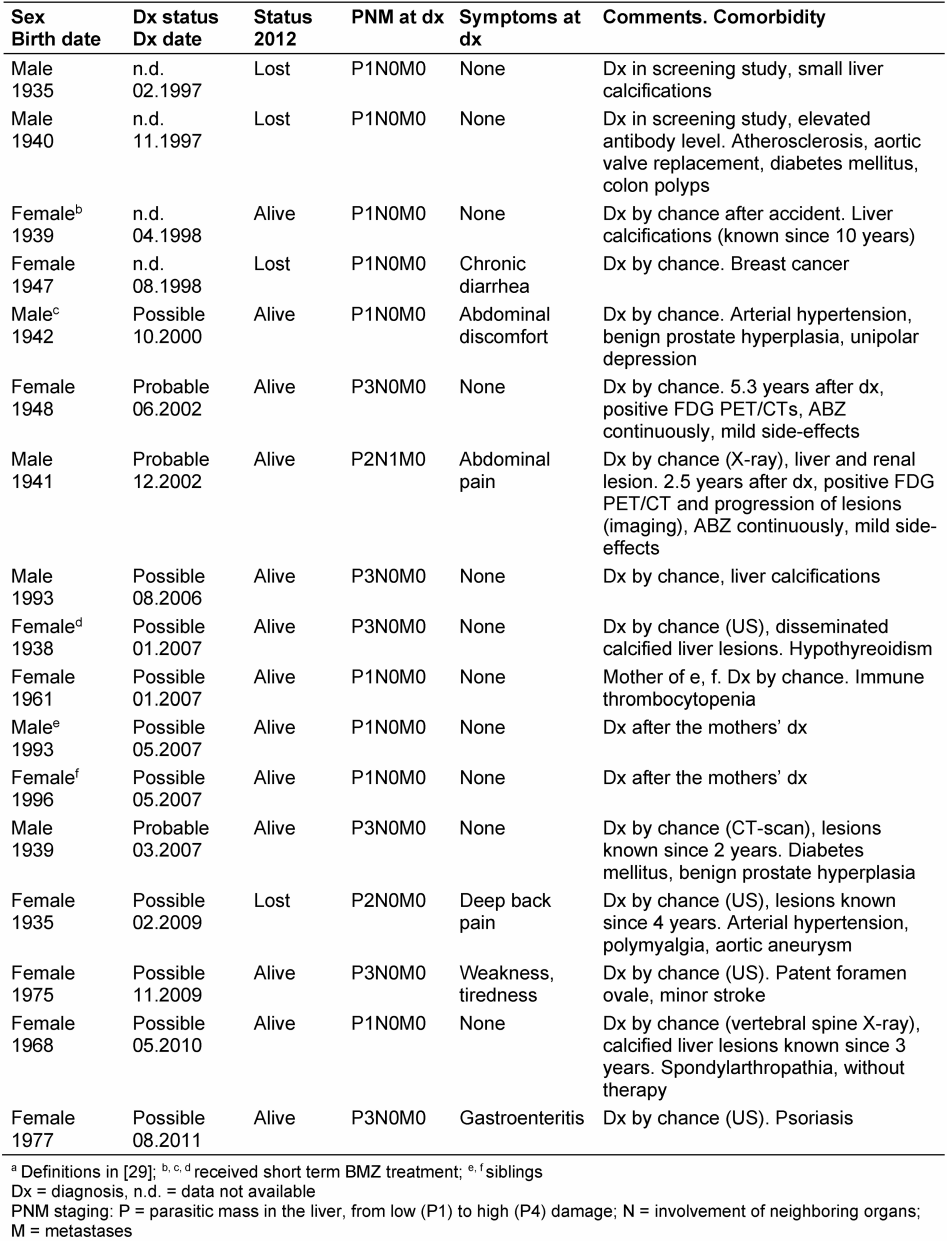
Patients with inactive lesions^a^ at diagnosis

**Table 5:**
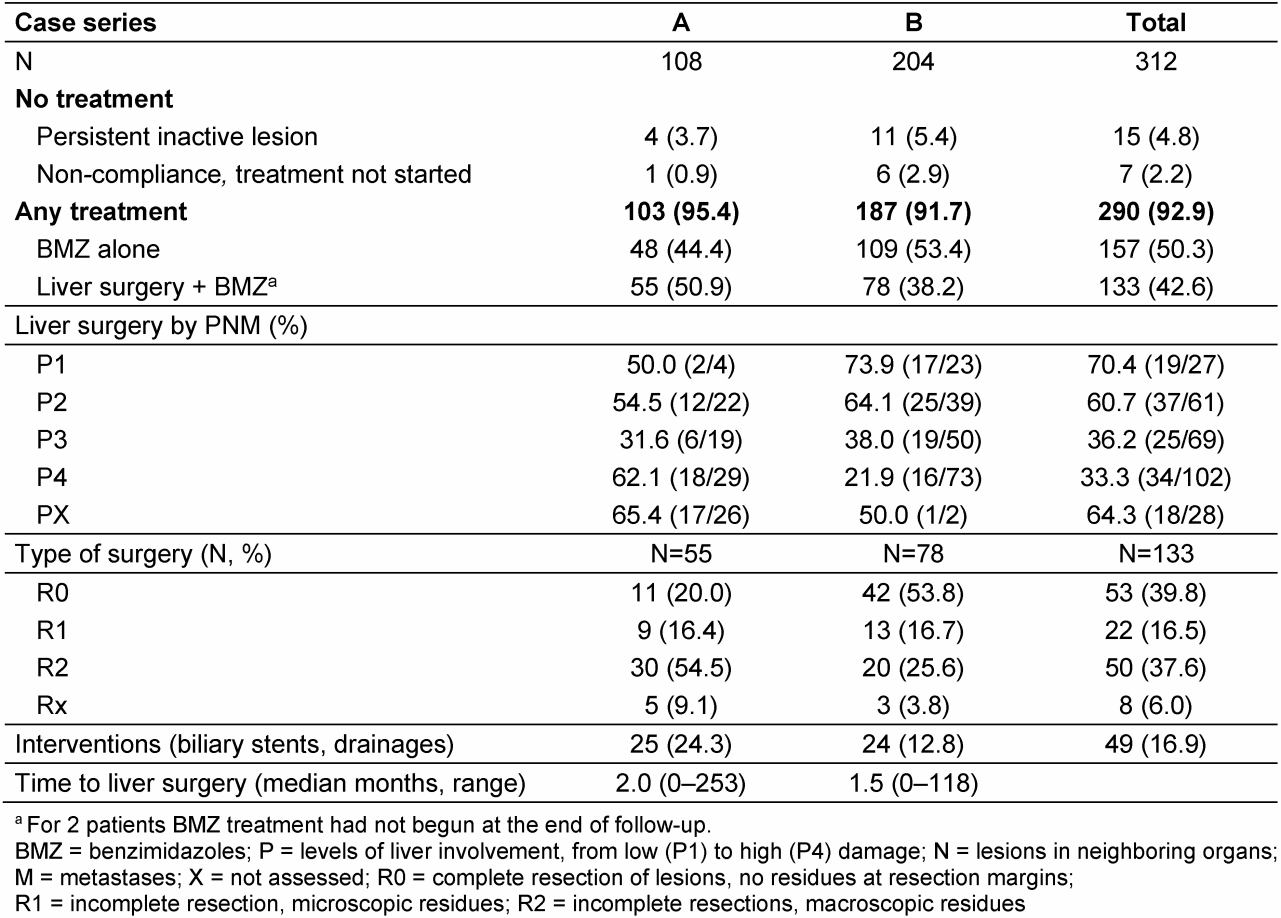
Treatment of alveolar echinococcosis

**Table 6:**
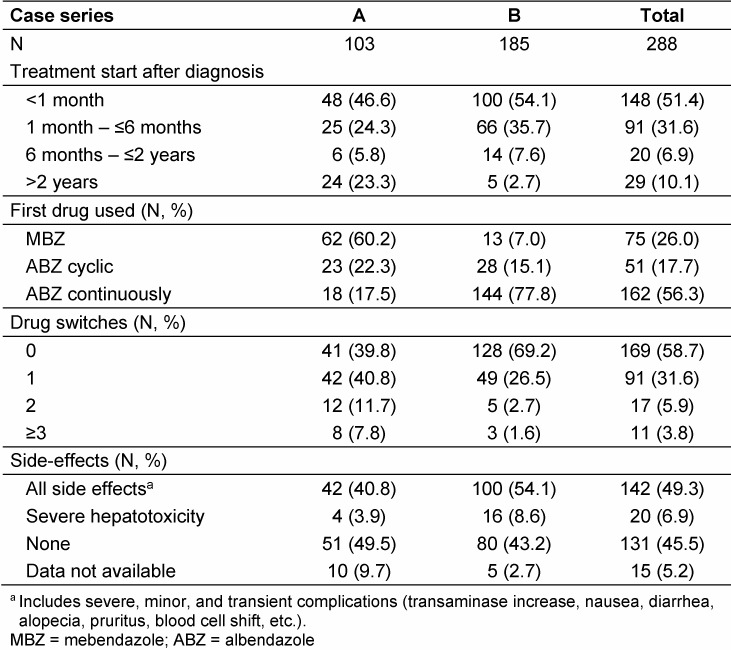
Benzimidazole treatment and side-effects

**Table 7:**
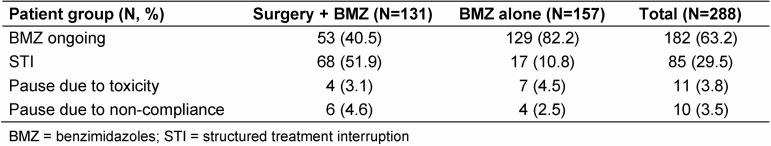
State of BMZ therapy at the end of follow-up

**Table 8:**
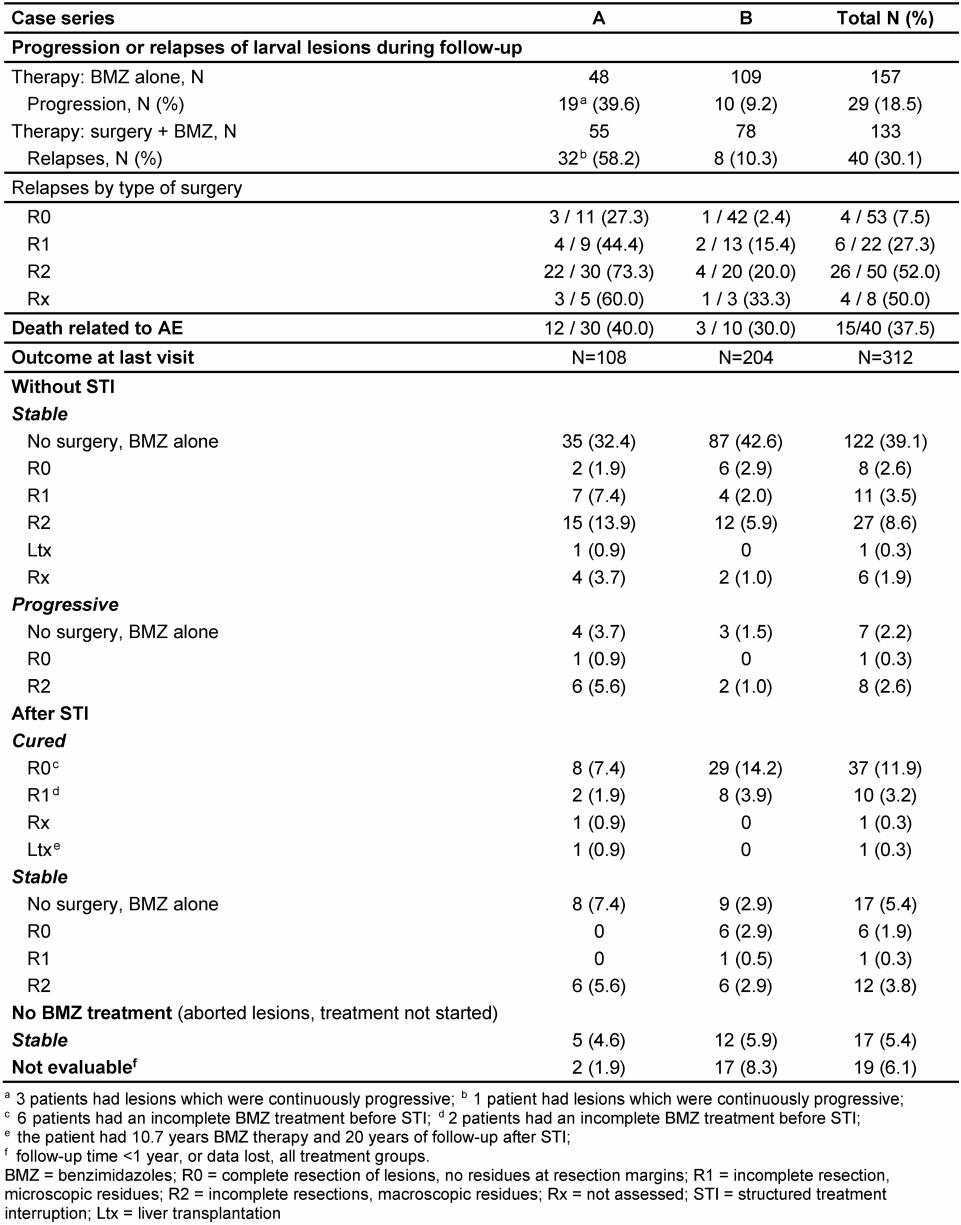
Outcome of patients with alveolar echinococcosis

**Table 9:**
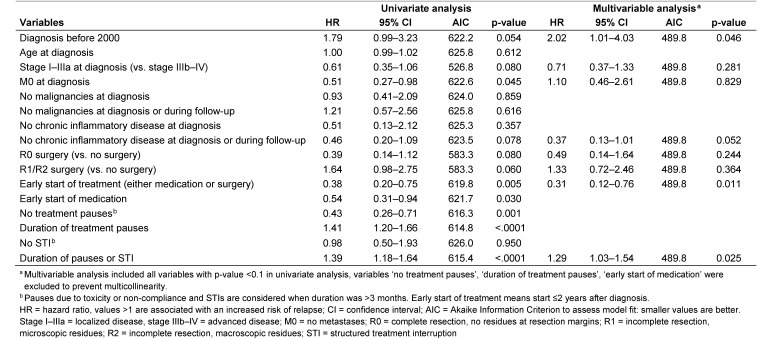
Cox proportional hazards regression analysis for covariates of relapse-free time
